# A comparison of two different techniques for the detection of blood parasite, *Theileria annulata*, in cattle from two districts in Khyber Pukhtoon Khwa Province (Pakistan)

**DOI:** 10.1051/parasite/2012191091

**Published:** 2012-02-15

**Authors:** R.M. Khattak, M. Rabib, Z. Khan, M. Ishaq, H. Hameed, A. Taqddus, M. Faryal, S. Durranis, Q.U.A. Gillani, R. Allahyar, R.S. Shaikh, M.A. Khan, M. Ali, F. Iqbal

**Affiliations:** 1 Department of Zoology, Kohat University of Science and Technology Kohat Pakistan; 2 Institute of Pure and Applied Biology, Zoology Division, Bahauddin Zakariya University Multan Pakistan; 3 Institute of Biotechnology, Bahauddin Zakariya University Multan Pakistan

**Keywords:** *Theileria annulata*, cattle, PCR amplification, smear scanning, Giemsa staining, risk factor, Khyber Pukhtoon Khwa, Pakistan, *Theileria annulata*, bétail, amplification par PCR, frottis, coloration de Giemsa, facteur de risque, Khyber Pukhtoon Khwa, Pakistan

## Abstract

The present study was carried out to determine the prevalence of *Theileria annulata* in large ruminants from two districts, Peshawar and Kohat, in Khyber Pukhtoon Khwa (Pakistan). Blood samples were collected from 95 cattle. Data on the characteristics of animals and herds were collected through questionnaires. No significant risk factors were found associated with the spread of tropical theileriosis in the study area. Two different parasite detection techniques, PCR amplification and screening of Giemsa stained slides, were compared and it was found that PCR amplification is a more sensitive tool (33.7% parasite detection), as compared to smear scanning (5.2% parasite detection) for the detection of *Theileria annulata*. 32 out of 95 animals, from both districts, produced the 721-bp fragment specific for *Theileria annulata*.

## Abstract (fr)

Une étude a été menée afin de déterminer la prévalence de *Theileria annulata* chez 95 bovidés de deux districts (Peshawar et Kohat) de la province de Khyber Pukhtoon Khwa au Pakistan. L’âge des bovins, la présence de tiques chez ceux-ci, ainsi que la présence de tiques chez les chiens du troupeau ne sont pas des facteurs de risque impliqués dans la diffusion de la theilériose dans la zone étudiée. La comparaison de deux techniques de détection du parasite (PCR et frottis sanguin coloré au Giemsa) a montré que la PCR était plus sensible (33,7 %) que le frottis (5,2 %).

*Theileria annulata* is a common protozoan parasite of cattle transmitted by *Hyalomma* sp. ticks, responsible for tropical theileriosis, a disease which has been reported from various parts of the world including Pakistan (Oliveira *et al.*, 1995; Durrani *et al.*, 2010; Tavassoli *et al.*, 2011). According to an estimation about 250 million cattle are threatened by this disease that acts as a major constraint on livestock production and improvement in many developing countries, which adversely affects economic performance, mainly by transmission of serious pathogens of animals (Uilenberg, 1981; Das *et al.*, 2005). The role of livestock in Pakistan’s rural economy may be estimated from the fact that 30-35 million rural population is engaged in raising livestock that gives rise to 30-40% of their income (Livestock Census, 1996).

*Theileria* sporozoites enter their bovine host during tick feeding and they rapidly invade mononuclear leukocytes, where they mature into macroschizonts and induce proliferation in host cells (Shahnawaz *et al.*, 2011). Microschizonts gradually develops into macroschizonts and ultimately into merozoites, which are released from leukocytes. These merozoites invade erythrocytes and develop into piroplasms. A marked rise in body temperature, reaching 40-41.5 °C is followed by depression, lacrimation, nasal discharge and swelling of the superficial lymph nodes, anemia are among the characteristic features of tropical theileriosis and as a result weight loss is rapid and haemoglobinuria may occur (Oliveira *et al.*, 1995; Gubbels *et al.*, 2000).

Advances in molecular biological techniques have resulted in the improved detection, identification and genetic characterization of many haemoparasites. Species-specific polymerase chain reaction (PCR) has been developed for the detection and identification of various *Theileria* species and has been shown to have higher sensitivity and specificity compared with serological assays and examination of Giemsa stained blood smears (Bhoora *et al.*, 2009; Shahnawaz *et al.*, 2011).

The aim of the present study was to establish and optimize a specific, reliable and sensitive molecular tool, the polymerase chain reaction (PCR), for the detection of *T. annulata* in cattle of Peshawar and Kohat districts of Khyber Pukhtoon Khwa and the comparison of results with parasite detection by Giemsa staining of blood smear. Furthermore, the present study provided a baseline regarding *T. annulata* prevalence and risk factors involved in the spread of tropical theileriosis in large ruminants.

## Materials and Methods

Blood samples were collected from 95 clinically healthy cattle from randomly selected herds, during November 2010 to February 2011, located in the two important livestock production regions, Peshawar and Kohat, in Khyber Pakhtoon Khwa Province of Pakistan. Blood was collected from the jugular vein of the animals and immediately preserved in Eppendorf tubes by adding a few drops of 0.5 M EDTA. Data regarding the characteristics of animals (species, gender, age, presence of ticks) and herd (location, size, dogs associated with the herds, presence of ticks on dogs associated with the herds) was collected through questionnaires completed by investigators on sampling sites in order to calculate the risk factors involved in the spread of tropical theileriosis.

Blood smears were prepared, fixed with methanol, stained with Giemsa and microscopically observed for the detection of *Theileria* sp. in blood.

Inorganic method of DNA extraction was used following Iqbal *et al.* (2011) and Zulfiqar *et al.* (2012). The quality of the DNA extract in regard to purity and integrity was assessed with optical density counts at 260/280 nm and submerged gel electrophoresis.

A set of oligonucleotide primers was used to amplify the 30-kDa gene sequences of *T. annulata* as previously described by Oliveira *et al.* (1995). The nucleotide sequence of the primer-pair was: (N516) 5’ GTAACCTTTAAAAACGT 3’ and (N517) 5’ GTTACGAACATGGGTTT 3’. PCR was performed in a final reaction volume of 25 μl. Each reaction contained 50 mM KCl, 10 mM Tris-HCl (pH 8.3), 1.5 mM MgCl_2_, 0.1% Triton X-100, 200 μM (each) deoxynucleotide triphosphate (dNTPs), 2.5 U of *Taq* DNA polymerase (Merck, USA), 20 pmol of primers and 5 μl of extracted DNA sample. *T. annulata* DNA (previously isolated from the blood of naturally infected cattle) and distilled water (without DNA) were also used, as templates in every PCR amplification, as positive and negative controls, respectively. DNA amplification was carried out in a thermal cycler (Gene Amp^®^ PCR system 2700 Applied Biosystems Inc., UK). The thermo-profile used by Oliveira *et al.* (1995) was modified for the present study. An initial denaturing step of five minutes at 94 °C was followed by five cycles: denaturing step of one minute at 94 °C, an annealing step of one minute at 56 °C and an extension step of one minute at 72 °C. These five cycles were followed by 30 cycles. Each cycle consisted of denaturing step of one minute at 94 °C, an annealing step of one minute at 54 °C and an extension step of one minute at 72 °C. The PCR program ended with a final extension step of seven minutes at 72 °C. Amplified products were separated by electrophoresis on a 1% agarose gel and visualized under a UV Transilluminator (Biostep, Germany).

Animals were grouped into two age categories: less than one year to one year (calf) and more than one year old (adult). Herds were divided into two size categories: herds having 1-15 animals and herds with more than 15 animals. The absence or presence of ticks on cattle, calf and dogs associated with the herds was also recorded. Association between the presence of *T. annulata* in cattle blood sample and the various parameters, *i.e.* herd location, herd size, species, gender and age of animal, and absence or presence of ticks on cattle, calf and dogs in the herd was assessed by contingency table analysis using the Fisher’s exact test (for 2 × 2 tables). All the values are expressed as mean ± standard deviations. Mini Tab (Version 16) was used for statistical analysis.

## Results

PCR results showed that 32 (33.7%) out of 95 examined blood samples, collected from two districts of Southern Punjab, produced the 721- bp fragment specific for *T. annulata*. The 32 parasite positive blood samples included 17 cattle samples from Kohat and 15 from Peshawar districts. On the other hand, only five (5.2%) of 95 blood samples were found parasite positive during microscopic examination of Giemsa stained blood smears. Prevalence of *T. annulata* was significantly (P = 0.053) higher in Kohat district as compared to Peshawar (Table I).

**Table I.** Sampling districts along with the total number of cattle samples collected (N) from each site. Prevalence of *Theileria annulata* is given in parenthesis. ANOVA results revealed a significant effect (P = 0.053) of location on parasite prevalence.

Sampling site	N	*T. annulata* + ive	*T. annulata* - ive
Peshawar	63	15 (23.8%)	48 (76.2%)
Kohat	32	17 (53.1%)	15 (46.9%)

Total animals	95	32 (33.7%)	63 (66.3%)

Statistical analysis of the characteristics of animals showed that age (P = 0.08) could play an important role in the spread of theileriosis as animals older than one year were more infected by the parasite, but this correlation did not reached statistical significance. Regarding the characteristics of herds, results indicated that size of herd and presence of ticks on dogs were not associated with the spread of theileriosis, as smaller herds composed of up to 15 animals (P = 0.18) and herds having dogs with ticks present on them (P = 0.82) were not more infested with the parasite (Table II).

**Table II.** Association between parasite prevalence in cattle and the studied parameters describing animal and herd characters.

Characters		Parameters	No. of Samples	T. annulata + ive	T. annulata - ive	P-value (F)
Animal	Sex	Male	9	1 (13%)	8 (87%)	0.26
		Female	86	31 (36%)	45 (64%)	(NS)
	
	Age	> 1 year	15	2 (15%)	13 (85%)	0.08
		< 1 year	80	30 (38%)	50 (62%)	(NS)
	
	Ticks	Absent	44	14 (32%)	30 (68%)	0.82
		Present	51	18 (35%)	33 (65%)	(NS)
	
	Prior treatment	Yes	4	3 (75%)	1 (25%)	0.10
		No	91	29 (32%)	62 (68%)	(NS)

Herd	Size	1–15 cattle	75	28 (37%)	47 (63%)	0.18
		More than 15 cattle	20	4 (20%)	16 (80%)	(NS)
	
	Dogs with herd	Absent	31	11 (36%)	20 (64%)	0.82
		Present	64	21 (33%)	43 (67%)	(NS)
	
	Ticks on dogs	Absent	35	11 (31%)	24 (69%)	0.82
		Present	60	21 (35%)	39 (65%)	(NS)

Probability of Fisher’s exact test is mentioned for each parameter. For all parameters, P > 0.05 and results are statistically non significant (NS).

## Discussion

Seven species of *Theileria* are known to infect cattle; of these, *T. annulata* is of major importance (Hasanpour *et al.*, 2008). It is difficult to differentiate *Theileria* species solely on the basis of the morphology of the piroplasm and schizont stages and confusion may arise if mixed infections occur (Dumanli *et al.*, 2005). There is a need to develop sensitive tools for the effective detection and treatment of *Theileria* sp. in order to decrease the economic losses by the parasite. In the present study, the major merozoite surface antigen gene of parasite (*Tams 1*) was amplified by PCR. *Tams 1* gene is the most abundant and immuno-dominant antigen on the surface of merozoites (asexual blood stage) of *T. annulata* having a molecular mass of approximately 30 kDa (Rady *et al.*, 2010). This gene has been targeted by several previous studies for the molecular detection of *T. annulata* in cattle and buffaloes (Oliveira *et al.*, 1995; Kirvar *et al.*, 1999; Dumanli *et al.*, 2005; Durrani *et al.*, 2010; Shahnawaz et al., 2011). The parasite was detected in samples from both districts under study and prevalence was higher in Kohat (53%) compared to Peshawar (24%) (Table I).

Several conventional and modern methods are used for the detection of *Theileria* sp. in host animals. The traditional one is by microscopic examination of blood smears stained with Giemsa. This technique is usually adequate for detection of acute infections, but not for detection of carrier animals, where parasitemia may be low (Altay *et al.*, 2008). Several studies documented that PCR is more specific and sensitive than conventional techniques for the detection of carrier ruminants having *Theileria* sp. present in blood without any apparent signs of theileriosis (Oliveira *et al.*, 1995; Martin-Sanchez *et al.*, 1999; Kirvar *et al.*, 1999). We have recently reported 19% prevalence of *T. annulata* in large ruminants, in six districts from southern Punjab, detected through PCR (Shahnawaz et al., 2011). The present study also confirmed the previous findings that PCR is more reliable technique of parasitic amplification as only five out of 95 blood samples were found to be parasite-positive by screening the Giemsa stained blood films. All the smearpositive samples were also picked by PCR amplification. Only microscopic examination of PCR positive samples would have declared them parasite free. A similar comparison was made by Durrani *et al.* (2010) in Sahiwal (Pakistan) and found 23% prevalence for *T. annulata* in cattle by PCR as compared to 5% prevalence detected by blood smear examination.

Analysis of data revealed that cattle more than 12 months old (38%) could be more infected by *T. annulata* compared to calves (15%) (P = 0.08). This result was contradictory, not only to our previous findings, but also with the findings of Qayyum *et al.* (2010), where a high incidence of *T. annulata* in calves compared to adult cattle in various livestock farms of Punjab was observed. This indicates that geographical and environmental conditions affects parasite prevalence confirming the findings of Dumanli *et al.* (2005). The presence of ticks on animals proved to be an important risk factor for the spread of theileriosis during the present study. Ticks were found on 35% of the infected animals (P = 0.02) suggesting that ticks were the vector for this parasite although this correlation was not statistically significant. A similar trend of tick infestation and theileriosis was previously reported by Rehman *et al.* (2004) and Ghosh *et al.* (2007).

The prevalence of *T. annulata* was not higher in small herds (37%) composed of up to 15 cattle than in large herds (20%) having more than 15 animals. A contradictory association between parasite prevalence and herd size was observed by Shahnawaz et al. (2011), who reported more parasite infestations in large herds. During the study, prevalence of the parasite was not higher in herds having tick-infested dogs (35%) than in herds having dogs without tick infestation (31%) (P = 0.82). Our results indicated that the prevalence of *T. annulata* is significantly higher than the previously reported in various parts of Pakistan. By following the risk factor assessment in this paper, the quality of livestock can be markedly improved in a province whose economy mainly based upon livestock.
